# Patent analysis of digital sensors for continuous glucose monitoring

**DOI:** 10.3389/fpubh.2023.1205903

**Published:** 2023-08-09

**Authors:** Olena Litvinova, Magdalena Eitenberger, Aylin Bilir, Andy Wai Kan Yeung, Emil D. Parvanov, ArunSundar MohanaSundaram, Jarosław Olav Horbańczuk, Atanas G. Atanasov, Harald Willschke

**Affiliations:** ^1^Department of Management and Quality Assurance in Pharmacy, National University of Pharmacy of the Ministry of Health of Ukraine, Kharkiv, Ukraine; ^2^Ludwig Boltzmann Institute Digital Health and Patient Safety, Medical University of Vienna, Vienna, Austria; ^3^Division of Oral and Maxillofacial Radiology, Applied Oral Sciences and Community Dental Care, Faculty of Dentistry, The University of Hong Kong, Hong Kong, China; ^4^Department of Translational Stem Cell Biology, Research Institute of the Medical University of Varna, Varna, Bulgaria; ^5^School of Pharmacy, Sathyabama Institute of Science and Technology, Chennai, Tamil Nadu, India; ^6^Department of Biotechnology and Nutrigenomics, Institute of Genetics and Animal Biotechnology of the Polish Academy of Sciences, Jastrzebiec, Poland; ^7^Department of Anaesthesia, Intensive Care Medicine and Pain Medicine, Medical University of Vienna, Vienna, Austria

**Keywords:** continuous glucose monitoring, minimally invasive sensors, non-invasive sensors, digital sensors, diabetes

## Abstract

The high need for optimal diabetes management among an ever-increasing number of patients dictates the development and implementation of new digital sensors for continuous glucose monitoring. The purpose of this work is to systematize the global patenting trends of digital sensors for continuous glucose monitoring and analyze their effectiveness in controlling the treatment of diabetes patients of different ages and risk groups. The Lens database was used to build the patent landscape of sensors for continuous glucose monitoring. Retrospective analysis showed that the patenting of sensors for continuous glucose monitoring had positive trend over the analyzed period (2000–2022). Leading development companies are Dexcom Inc., Abbott Diabetes Care Inc., Medtronic Minimed Inc., Roche Diabetes Care Inc., Roche Diagnostics Operations Inc., Roche Diabetes Care Gmbh, and Ascensia Diabetes Care Holdings Ag, among others. Since 2006, a new approach has emerged where digital sensors are used for continuous glucose monitoring, and smartphones act as receivers for the data. Additionally, telemedicine communication is employed to facilitate this process. This opens up new opportunities for assessing the glycemic profile (glycemic curve information, quantitative assessment of the duration and amplitude of glucose fluctuations, and so on), which may contribute to improved diabetes management. A number of digital sensors for minimally invasive glucose monitoring are patented, have received FDA approval, and have been on the market for over 10 years. Their effectiveness in the clinic has been proven, and advantages and disadvantages have been clarified. Digital sensors offer a *non-invasive* option for monitoring blood glucose levels, providing an alternative to traditional invasive methods. This is particularly useful for patients with diabetes who require frequent monitoring, including before and after meals, during and after exercise, and in other scenarios where glucose levels can fluctuate. However, non-invasive glucose measurements can also benefit patients without diabetes, such as those following a dietary treatment plan, pregnant women, and individuals during fasting periods like Ramadan. The availability of non-invasive monitoring is especially valuable for patients in high-risk groups and across different age ranges. New world trends have been identified in the patenting of digital sensors for non-invasive glucose monitoring in interstitial skin fluid, saliva, sweat, tear fluid, and exhaled air. A number of non-invasive devices have received the CE mark approval, which confirms that the items meet European health, safety, and environmental protection standards (TensorTip Combo-Glucometer, Cnoga Medical Ltd.; SugarBEAT, Nemaura Medical; GlucoTrack, GlucoTrack Inc.), but are not FDA-approved yet. The above-mentioned sensors have characteristics that make them popular in the treatment of diabetes: they do not require implantation, do not cause an organism reaction to a foreign body, and are convenient to use. In the EU, in order to increase clinical safety and the level of transparency about medical devices, manufacturers must obtain certificates in accordance with Regulation (EU) 2017/745, taking into account the transition period. The development of systems, which include digital sensors for continuous glucose monitoring, mobile applications, and web platforms for professional analysis of glycemic control and implementation of unified glycemic assessment principles in mobile healthcare, represent promising approaches for controlling glycaemia in patients.

## 1. Introduction

According to statistics from the International Diabetic Federation, as of 2021, diabetes mellitus (DM) has higher prevalence in developed countries due to technical progress, urbanization and changes in the life style and diet, that lead to obesity and high blood pressure. The prevalence of Type 1 diabetes mellitus (DM1) varies across different regions and populations. The most prevalent form of diabetes, type 2 (DM2), affects more than 90% of diabetic people globally. The increasing life expectancy has led to a rise in the incidence of DM. If such trends persist, then by 2045 this figure will grow to 783 million adults aged 20–79 years (1).

According to the World Health Organization (WHO), diabetes mellitus is among the top 10 leading causes of death worldwide. The prevalence of the disease is rising, with a notable trend of affecting individuals at younger ages.

Achieving optimal and sustained glycemic control is one of the most effective approaches to prevent complications and reduce mortality in patients with DM. Glycemic variability is a key therapeutic focus in the treatment of diabetic patients (2–5).

Continuous glucose monitoring (CGM) is crucial in achieving glycemic control for most patients with DM1 and DM2 in accordance with the American Diabetes Association's Standards of medical care in diabetes (6).

CGM is strongly recommended by the International Society for Pediatric and Adolescent Diabetes Guidelines for all children, adolescents, and young adults with DM1 (7).

The market of innovative medical products for the treatment of DM1 is constantly expanding, namely with the development of insulin pumps, closed-loop systems. The first model of a hybrid closed loop system (the MiniMed 770G System) for patients aged 2 to 6 with DM1 was approved by the FDA in 2020 (8).

The clinical accuracy of continuous glucose monitoring in patients with DM1 is shown in systematic reviews of randomized trials and meta-analyses by Teo et al., Moser et al., and Elbalshy et al. (9–11). It is emphasized that future research should assess the precision and efficiency of CGM for various age groups and insulin therapy regimens. Abrupt changes in glucose levels were associated with lower measurement accuracy. Wang et al. and Daskalaki et al. also noted that CGM may benefit diabetic patients (12, 13).

Mihaya et al. and Fedorova et al. compared the different methods of CGM and readers are referred to these previous works for more detailed comparative overview. The authors also reviewed different classifications of sensors for continuous glucose measurement, including by analytical methods (14, 15).

In reviews by Lin and Gonzales et al., much attention is paid to the technical characteristics of sensors for CGM (16, 17). Data on the applied technologies and measurement accuracy parameters are presented. Unfortunately, no validation in patients or volunteers is described. Shang et al. have characterized products for continuous monitoring of glucose levels based on their consumer, technological, and regulatory features (18).

Underscoring the economic side of the treatment of diabetes mellitus, researchers draw attention to the fact that, at present, direct costs for the treatment of diabetes are high and reach about 1 trillion US dollars; by 2030, they are predicted to double. At the same time, there is evidence that continuous glucose monitoring has both positive clinical and economic effects in patients with diabetes (19).

While the necessary methodological basis exists, at the same time, many aspects of the application of continuous glucose monitoring by digital sensors need additional research and clarification.

It has to be mentioned that the measurement of glucose by non-invasive methods has several limitations. CGM is dependent on skin properties, changes in the blood supply of tissue, concomitant therapy affecting the distribution of fluid in tissues, concomitant diseases, skin edema, hypotension, etc. (20–24). Additionally, the requirement for capability to detect slight variations in glucose levels during continuous monitoring needs to be taken into consideration.

Thus, at the moment, scientific research covers a wide range of aspects with relevance for CGM. However, there are still insufficiently explored issues related to minimally invasive and non-invasive continuous glucose monitoring in people of different ages and risk groups, respective patent protections, and implemented digital technologies. The increase in the requirements for modern medical digital systems contributes to the active search for medical sensor technologies for continuous glucose monitoring, combining simultaneously informational, analytical, predictive, and control functions.

Digital technologies for people with diabetes are becoming an increasingly common aspect of monitoring the effectiveness of diabetes care (25, 26). Further development and implementation of mobile applications and web platforms for continuous glucose monitoring devices is a promising approach.

Patent information concerning innovative technologies appears in patent databases earlier than in scientometric databases the market introduction. Using an analysis of the global patent landscape, researchers can identify the most active and promising areas of research and direct their resources accordingly.

The purpose of this work is to systematize the global patenting trends of digital sensors for continuous glucose monitoring and gain insights on their effectiveness in controlling the treatment of diabetes patients of different ages and risk groups.

## 2. Materials and methods

The information base for building the patent landscape was compiled by the Lens database as of *December 31, 2022*. During the study, we used the following methods: system and logical analysis, synthesis, methods of comparison, graphic representation methods, patent research, content analysis, and others.

**Figure 1:**
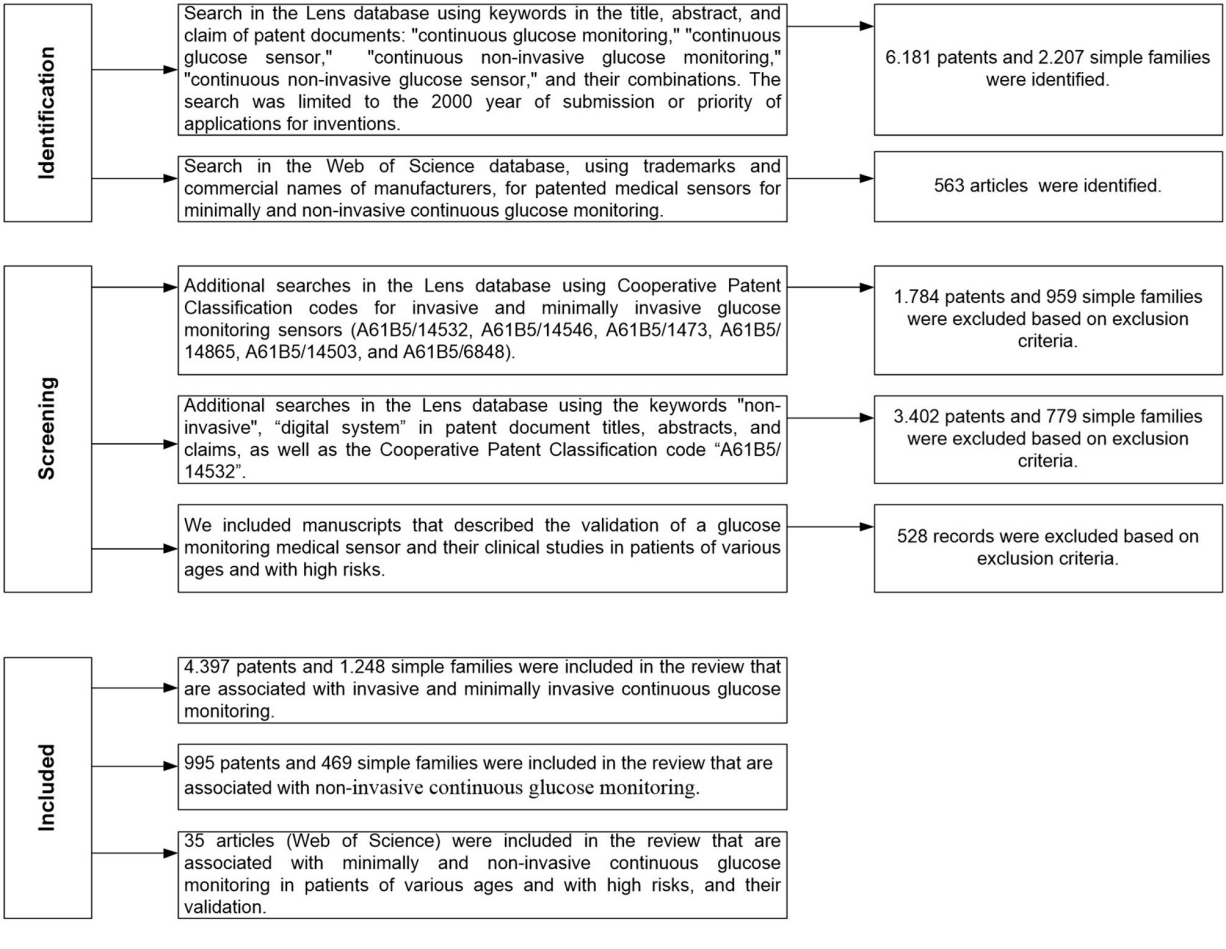
The strategy of patent and literature studies.

The strategy of the conducted patent and literature studies is shown in Figure 1.

Key words selected as search queries are “continuous glucose monitoring,” “continuous glucose sensor,” “continuous non-invasive glucose monitoring,” “continuous non-invasive glucose sensor,” “non-invasive glucose,” “non-invasive sensor,” “digital system,” and their combinations. A search by keywords was carried out in the title, abstract, and claim of patent documents.

Patent classification codes were also used in the search. It should be noted that the classification of inventions, including in the field of continuous glucose monitoring, is carried out by specific experts of the patent office, i.e., it is largely subjective, which explains the wide spread of patents by class in the International Patent Classification and the Cooperative Patent Classification. In addition, various analytical methods are used for continuous glucose monitoring, which also leads to the use of a wide range of patent classification codes. The study used the code of the Cooperative Patent Classification “A61B5/14532,” which is the most widely used by leading manufacturers and also covers various analytical approaches. We also used codes from the Cooperative Patent Classification, which are connected with invasive, minimally invasive, and non-invasive sensors (A61B5/14546, A61B5/1473, A61B5/14865, A61B5/14503, and A61B5/6848) (27). For example, the code of the Cooperative Patent Classification “A61B5/14865” is related to the measurement of glucose using implanted sensors.

The search was limited to inventions submitted or prioritized since the year 2000. Using the in-build Lens database's functionality, a numerical analysis of the found documents was done.

As a rule, the presence of patent protection for a technology in the territory of a country indicates the presence of potential demand for it. An analysis of inventive activity regarding geography for patent coverage and the patent offices was carried out.

In the Web of Science database, clinical studies of sensors for minimally and non-invasive continuous glucose monitoring were analyzed using their trademarks and commercial names of manufacturers as keywords.

Exclusion criteria were used to narrow the search results. Irrelevant patents were excluded using the Cooperative Patent Classification codes. The excluded manuscripts were reviews of previous literature and manuscripts related to invasive glucose monitoring technologies.

The FDA medical device database (https://www.accessdata.fda.gov/scripts/cdrh/cfdocs/cfRL/rl.cfm) and the European database on medical devices “Eudamed” (https://ec.europa.eu/tools/eudamed/#/screen/home) were used to examine the registration and certification of patented sensors for minimally and non-invasive continuous glucose monitoring in accordance with ISO standards.

## 3. Results

### 3.1. Current trends in patenting within the segment of continuous glucose monitoring

**Figure 2:**
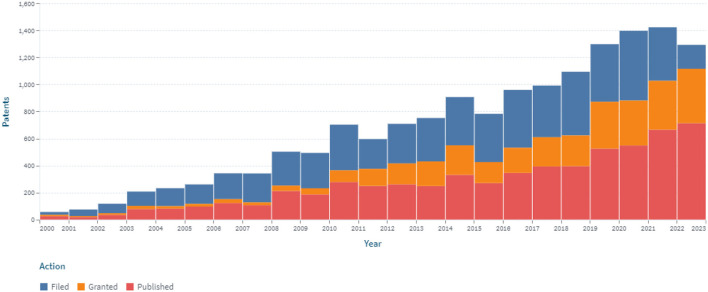
The dynamics of the patent landscape within the segment of continuous glucose monitoring in the period 2000–2022.

Patent analysis has identified 6.181 patents and 2.207 simple families in the area of continuous glucose monitoring filed between 2000 and 2022 (*accessed on December 31, 2022*; Figure 2).

A stable increase in the number of patents over the period 2000–2022 was identified, which is associated with the development of new sensors for CGM (Figure 2). At the same time, the largest number of patents were published in 2018 and 2020, covering 7.6 and 8.4% of all publications, respectively, for 2000–2022. The number of patents in 2015, 2016, and 2017 compared to 2005 increased by 2.48, 2.97, and 2.73 times, respectively. Retrospective analysis showed that the advancement of technologies for sensors for continuous glucose monitoring had a positive trend during the analyzed period. At the same time, the largest (516) number of applications was submitted in 2020.

**Figure 3:**
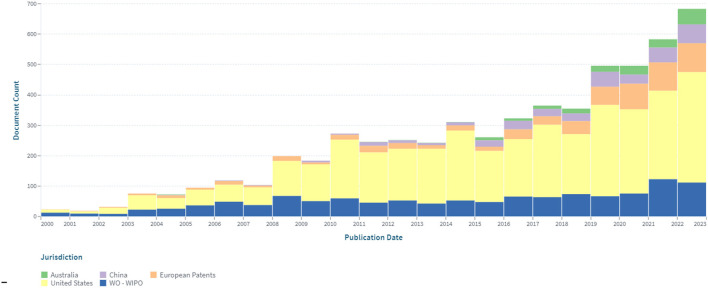
The dynamics of patenting in five leading patent offices within the segment of continuous glucose monitoring in the period 2000–2022.

An analysis of the patenting of sensors for continuous glucose monitoring by the patent offices was also carried out. Figure 3 depicts the dynamics of their patenting in five leading patent offices.

The largest number of patents was registered in the United States (3.482), which amounted to about 56% of the total data set. A significant (19%) share of patents related to continuous glucose monitoring are international documents filed under the PCT procedure. Advantages of this procedure are that: by filing at the initial stage of the application in one language, according to the results of an international search, researchers can choose a circle of patenting countries, obtaining a strong patent that is difficult to dispute. Under the PCT (patent cooperation treaty) system, it is economically feasible to patent the invention in more than five countries. The number of patents (602), conducted through the European Patent Office, is 9.7% of the total number.

**Figure 4:**
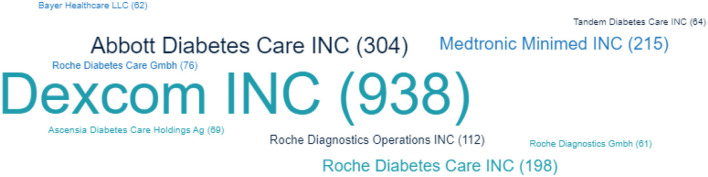
Top companies (by number of patents, reflecting the presented letter size) in the patenting field of devices for continuous glucose monitoring in the period 2000–2022.

The leaders in the patenting field of devices for continuous glucose monitoring are Dexcom Inc., Abbott Diabetes Care Inc., Medtronic Minimed Inc., Roche Diabetes Care Inc., Roche Diagnostics Operations Inc., Roche Diabetes Care Gmbh, Ascensia Diabetes Care Holdings Ag, etc. (Figure 4). Their patented inventions are covering invasive, minimally invasive, and non-invasive devices.

It should also be noted that start-up companies are also creating innovative technologies for glucose monitoring. For example, the GWave sensor is a device about a third the size of a standard smartphone, inserted into a ceramic bracelet. It uses Bluetooth to transmit its glucose readings to an accompanying mobile app that tracks readings and alerts users to fluctuations in their blood sugar levels (28).

**Figure 5:**
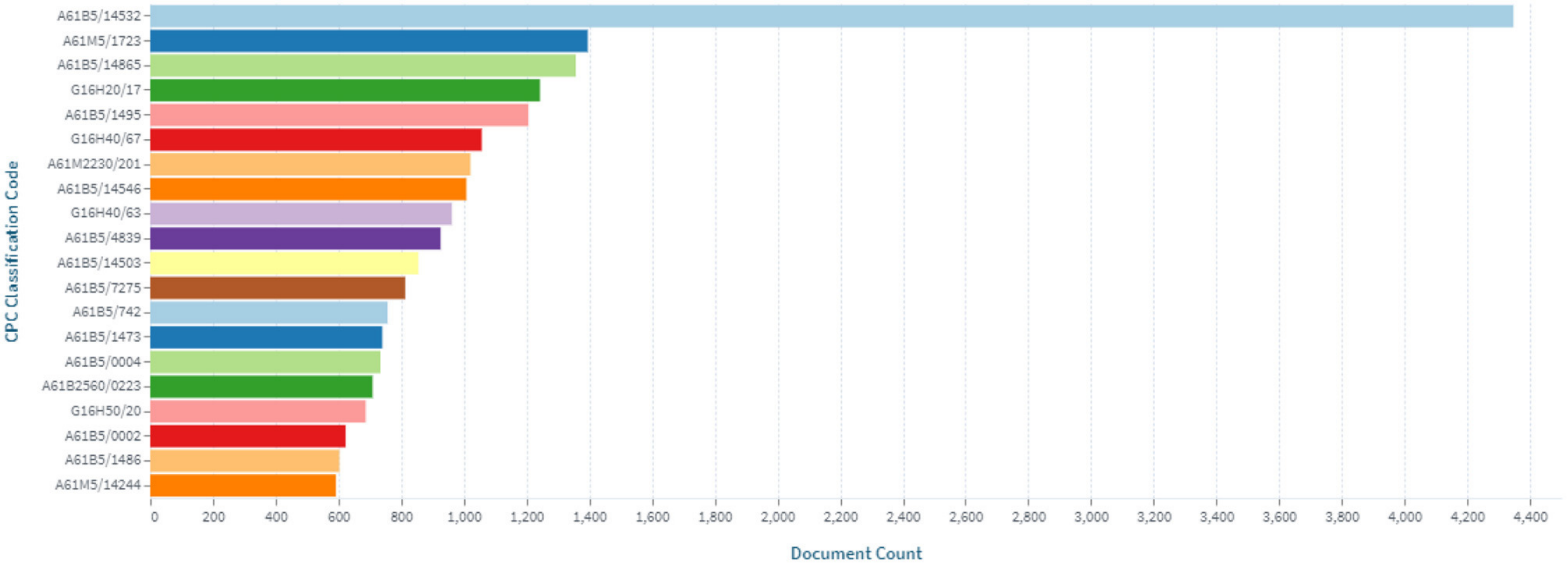
Distribution of inventions in the field of continuous glucose monitoring by Cooperative Patent Classification codes.

The inventions in the field of continuous glucose monitoring devices are most related to the Cooperative Patent Classification codes, which are shown in Figure 5.

Currently, on the background of recent ubiquitous technological developments penetrating the society, using of digital technologies to control the treatment of patients, including those with diabetes, and enhance their quality of life is becoming more relevant.

**Figure 6:**
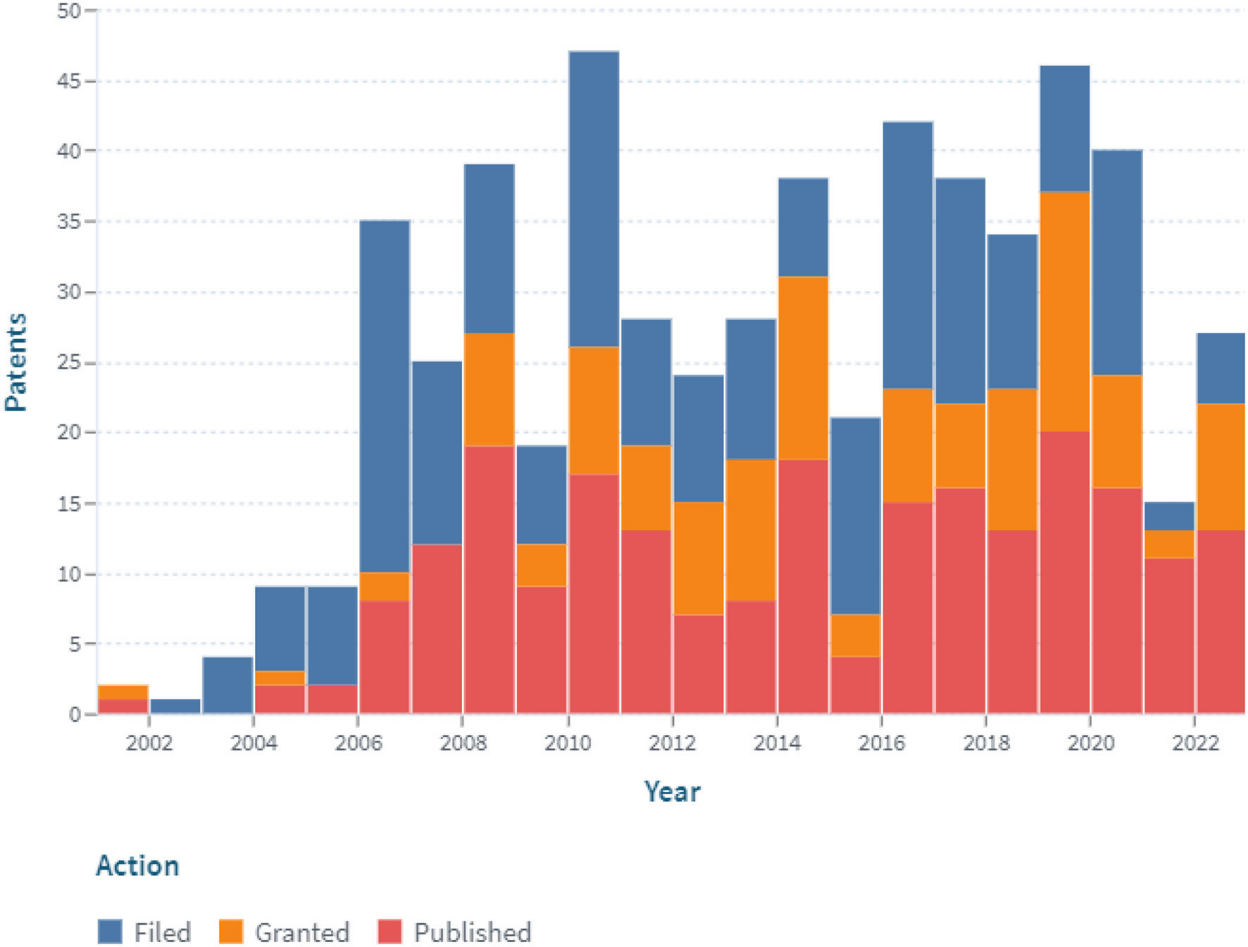
The dynamics of the patent landscape within the segment of digital continuous glucose monitoring in the period 2000–2022 (with the application of the additional keyword “digital system”).

A patent analysis using the additional keyword “digital system” revealed that since 2006, the number of claimed and issued patents associated with the digitalization of constant glucose monitoring has been increasing (Figure 6).

In the past 2 years (2021 and 2022), the number of filed patent applications has dropped significantly. On the one hand, this may be due to the increased efforts of inventors in the fields of prevention and treatment of COVID-19, development of insulin pumps and closed-loop systems, etc. On the other hand, it could be related to the bringing of previously patented technologies to market.

The implementation of digital technologies for continuous glucose monitoring increases its usability and provides new options for assessing the glycemic profile, such as information on the trend of the glycemic curve, quantification of the duration and amplitude of glucose fluctuations.

The devices for CGM are also divided into the following types: continuous glucose monitoring in the “blind” regime, real-time continuous glucose monitoring, as well as scanning continuous glucose monitoring (flush glucose monitoring) (29).

The sensor is placed for several days for continuous glucose monitoring in the “blind” regime. Following that, the obtained outcomes are retrospectively evaluated. The goal of monitoring is to detect changes in glycemia in the subject's everyday life settings. The sensor does not send any indications indicating a fall or increase in glycemia, allowing researchers to rule out as much “increased motivation” and “false compensation” of carbohydrate metabolism throughout the study time as is feasible. Real-time continuous glucose monitoring shows a detailed graph of glycemia variations over time and alerts when glycemia levels exceed individual objectives. Flash glucose monitoring is a technology in which patients scan information from a sensor that continuously monitors and stores glucose data for the last few hours, with the ability to view previously stored values.

Modern developments for continuous glucose monitoring are aimed at improving the accuracy and usability of sensors. Thus, it should be noted that innovative devices for constant glucose monitoring not only display a graph of the change in the amount of glucose in the blood but also warn the patient when this indicator is out of normal range and are also synchronized with the mobile application for smartphones.

According to ISO 15197:2015, the sensor is considered accurate if 95% of the test results are within ±0.83 mmol/L (±15 mg/dL) at glucose concentrations of < 5.55 mmol/L (< 100 mg/dL). At higher glucose concentrations, 95% of test results should be within ±15%. But this standard applies to glucometers. Most CGM accuracy assessment methods are based on comparing the CGM tool with the corresponding reference values.

The Clarke error grid analysis can be used to evaluate the clinical precision of a medical sensor for continuous glucose monitoring (30) as follows:

Zone A: Clinical decisions based on these indicators will result in identical results to those based on values obtained by the reference method.
Zone B: The indicators will not lead to an error in the prescription of treatment, or the error will be insignificant and will not affect the patient's condition.
Zone C: The use of the obtained indicator will lead to a serious error, which will most likely worsen the patient's condition.
Zone D: The score will lead to a very serious error, which will greatly worsen the patient's condition.
Zone E: The use of such an indicator will result in a fatal error that may be life-threatening to the patient.

The mean absolute relative difference (MARD) between devices is another measure for evaluating the accuracy of CGM systems. Valid MARD values require a significant number of paired measurements and are also related to the precision of the reference tool.

The clinical accuracy of continuous glucose monitoring is also assessed using Parkes nomograms (31). Zone A in the Parkes nomogram indicates no effect on clinical action. Zone B means changes in clinical action that have no or little effect on clinical outcome. Results in zones C, D, or E mean changes in clinical action that have a major impact on clinical outcome.

We systematized data on the world patent protection of invasive, minimally invasive, and non-invasive sensors. Minimally and non-invasive sensors for continuous glucose monitoring are of particular interest. Analysis was performed for clinical studies in patients of various ages and with high risks, and their implemented digital technologies, and data on their validation and registration by legislative bodies were summarized.

### 3.2. Invasive and minimally invasive continuous glucose monitoring

**Figure 7:**
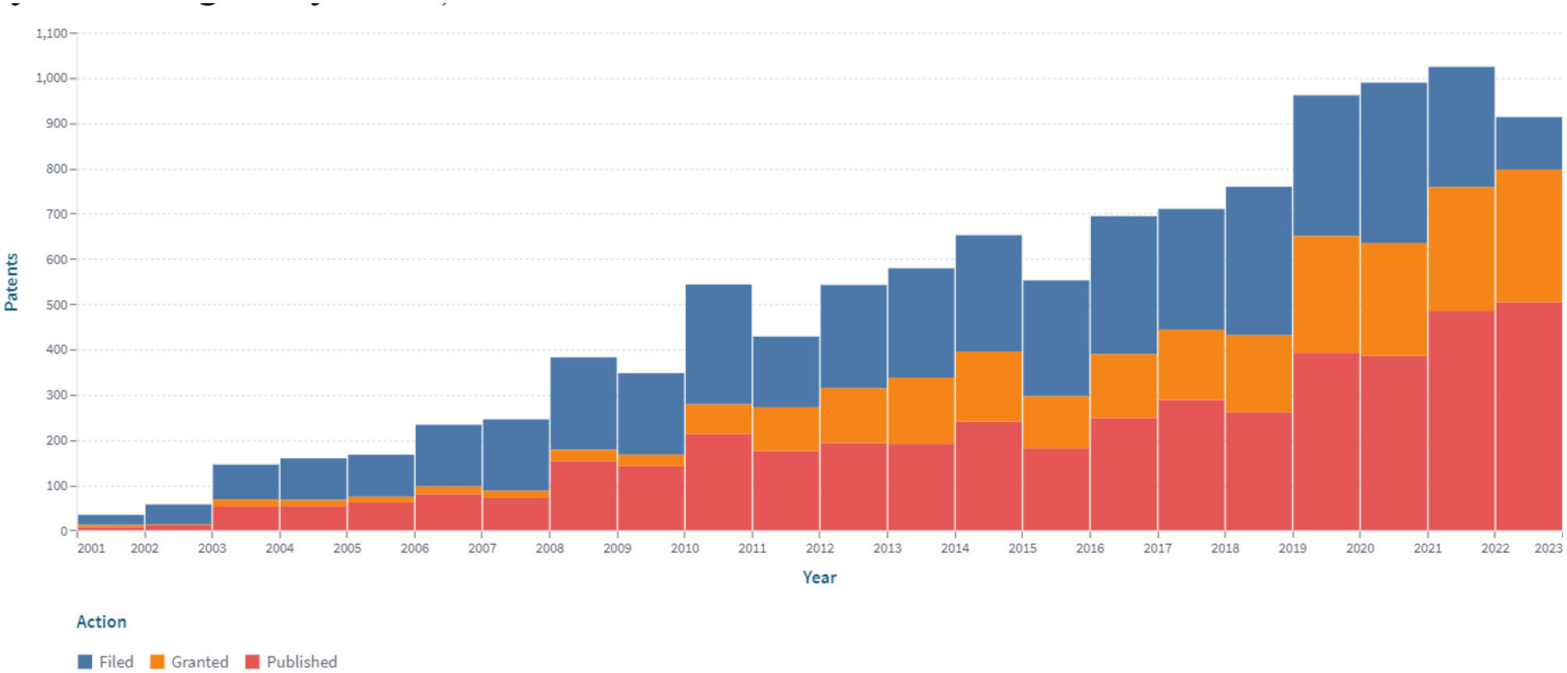
The dynamics of the patent landscape within the segment of invasive and minimally invasive continuous glucose monitoring in the period 2000–2022.

An additional filter with codes from the Cooperative Patent Classification was added to analyze the technological solutions associated with invasive and minimally invasive sensors for glucose monitoring (A61B5/14532, A61B5/14546, A61B5/1473, A61B5/14865, A61B5/14503, and A61B5/6848). The obtained data are shown in Figure 7. According to the conducted research, there are 4.397 patents and 1.248 simple families. The linear increasing of the patenting process has been revealed. These developments may also be presented with other patent classification codes and keywords.

The most patent publications concern obtaining patent protection in the United States (2.559; 58%), under the PCT procedure (711; 16%), and through the European Patent Office (473; 10.7%).

The first developments for continuous minimally invasive glucose monitoring in the “blind” regime include the 1999 invention by Medtronic MiniMed, USA. This device was able to measure the concentration of glucose in the interstitial fluid of subcutaneous fat every 5 min for 3 days in a row, after which the data was transferred to a computer and analyzed.

Of note is the Guardian Real-Time (Medtronic MiniMed, US) continuous minimally invasive monitoring device, which has been used for more than 15 years in clinical practice since 2004. It is capable of displaying the level of glucose in the blood in real time. The device is protected by numerous patents (US6809653, AU5623100, CA2666429, etc.) and measures glucose levels in interstitial fluid (32–34). It is necessary to calibrate the glucose concentration in the blood when using the device. The sensor is replaced after a few days (72 h). Medtronic has created a pediatric version of the Guardian Real-Time system specifically for young diabetics, which is quite remarkable.

There is evidence from a study on the effectiveness of implementing the Guardian Real-Time system in a clinical setting. This system is effective in children and adults against the background of insulin injections and insulin pump therapy for DM1 and DM2, as well as for heart surgery in children (35–39). Researchers noted a greater decline in HbA1c when using Guardian Real-Time monitoring. The Guardian real-time device's inaccuracies have been linked to abrupt changes in glucose levels in athletes (40). In addition, 56% measurement accuracy was observed in the hypoglycemic state.

It should be noted that Medtronic MiniMed develops new generations of devices and improves sensors for continuous glucose monitoring. An important fact is that Medtronic has gotten a CE mark for its Guardian Connect mobile continuous glucose monitor and app. Data can be transferred from a mobile phone to the Medtronic CareLink web platform. Modern systems, which include digital sensors for CGM, mobile applications, and web platforms, are opening up new opportunities in the management of diabetes mellitus.

Also, the real-time continuous glucose monitoring systems of Dexcom, Inc., and Abbott Diabetes Care are widely used. The Dexcom G4 Platinum system is disclosed in numerous patents (including CA2664528, US7935057, AU8088601, US8073519, US8858434, US7771352, US8682408, and US9750460) (41–45).

But in 2023, Dexcom G4 is no longer available on the market.

Dexcom Inc. is constantly expanding its portfolio of solutions regarding continuous glucose monitoring, which includes the Dexcom G6, Dexcom G7, and Dexcom ONE systems, among others. A crucial area for the advancement of medical devices for continuous glucose monitoring is related to their compatibility with mobile devices, cellphones. For example, the Dexcom G5 mobile system is disclosed in US Patent 9020572 (46). Also, data can be transferred from a mobile phone to the Dexcom Clarity web platform. As noted, systems, which include digital sensors for CGM, mobile applications, and web platforms, are playing an increasing role in the management of patients with diabetes mellitus.

The Dexcom G7 provides accurate, single-digit MARD glucose readings clinically proven when placed on the arm or abdomen in adults with diabetes (47). The accuracy of the Dexcom G7 sensors placed on the arm or abdomen is similar to the accuracy of the Dexcom G5 and Dexcom G6 sensors placed on the abdomen (48). Task analysis required for successful insertion of sensors favors Dexcom G7 over earlier systems. The G7 CGM placed on the arm or abdomen was accurate in children and adolescents with DM1 (49). However, accuracy measurements in the younger cohort (2–6 years old) were relatively few (50). The G7 CGM system has a lower cognitive burden than earlier CGM systems for older people utilizing it and for trained diabetes care and education specialists (51).

In 2018, the FDA approved the first CGM system, Eversense (Senseonics, Inc.), with a fully implantable sensor to measure glucose for up to 90 days. The measured glucose levels are sent to a device-specific mobile app every 5 min (52). The CGM system Eversense is disclosed in patent US9901293 (53). Inventors use the fluorescence method for glucose monitoring.

In 2014, the Flash monitoring device, FreeStyle Libre (Abbott Diabetes Care, USA), was launched on the European market. The system uses an electrochemical sensor that fits on the back of the shoulder for up to 14 days and a FreeStyle Libre scanner. The system estimates the concentration of glucose in the intercellular fluid every minute. Glycemia data is displayed as the scanner reaches the implantable sensor. The device displays the history of values over the past 8 h as well as trends in glucose levels. The device can be used to retrospectively assess glucose levels. The device also has powerful patent protection (for example, EP1466156, US7620438, US7920907, EP1942801, AU2012271333, etc.) (54–58). FreeStyle Libre also performs calibration using the built-in glucometer. It is recommended to replace the sensor after 120 h.

The results of the studies confirm that the use of the FreeStyle Libre Flash device contributed to a decrease in the level of HbA1 in diabetic patients (59–61). FreeStyle Libre was more accurate in patients with moderate to rapid glucose changes compared to Dexcom G4 Platinum (62). However, real-time continuous glucose monitoring (rtCGM) was more effective than intermittently scanned continuous glucose monitoring for a number of parameters in patients with DM1 and very high risk (63). The use of FreeStyle Libre Flash device had some limitations. In children without diabetes who are overweight or obese, FreeStyle Libre Flash did not accurately assess glucose levels (64). Although the accuracy of the FreeStyle Libre was clinically acceptable during post-prandial rest and walking in overweight or obese young adults (65). The possibility of using FreeStyle Libre Flash during dietary treatment of patients without diabetes, during Ramadan in patients with DM1, and in pregnant women with DM1 and DM2 is shown (66–68). In addition, the successful use of FreeStyle Libre Flash by children with DM1 during summer camp is reported (69). Since 2017, the FreeStyle Libre system has a CE designation for use in diabetic pregnant women. Abbott has also launched new, improved systems for continuous glucose monitoring, FreeStyle Libre 2, FreeStyle Libre 3.

In addition to the continuous glucose monitoring system, Abbott has also created a single digital ecosystem that includes the FreeStyle LibreLink and LibreLinkUp smartphone apps as well as the LibreView cloud platform. The FreeStyle LibreLink application (patents US7826382, US 10820842, etc.) allows patients to measure their glucose level, evaluate the data obtained, and share it with specialists (70, 71). In addition, the LibreLinkUp application (patent US11147479) enables patients to share their glucose data with relatives and friends for their peace of mind (72). LibreView (patents US10872696 and US10923218) is a free and secure cloud system with a web interface through which health workers and patients can create and share a set of structured, understandable reports (73, 74). The doctor installs LibreView on his computer. The patient transfers data from the FreeStyle LibreLink mobile app to the LibreView patient account. With the consent of the examined, physicians can view remotely downloaded patient glucose scores on their professional LibreView account. The doctor works with patient data, compiling reports at anytime and anywhere remotely where there is access to the Internet. The report is generated for any period of interest, and if necessary, the data is evaluated over time.

Abbott's digital ecosystem, like other CGM systems available on the market, provides doctors and patients with continuous online access to glycemic profile data, as well as the ability for additional patient support from their relatives or patient caregivers. It is able to analyze results over time, adjust therapy, and eliminate errors made by patients.

### 3.3. Non-invasive continuous glucose monitoring

For characterization of the patent landscape, not only widespread technologies are of importance, but also narrow segments that have evolved in recent years. Such important recently evolved segment is the one encompassing non-invasive continuous glucose monitoring technologies.

Many methods from various branches of biophysics have been proposed for assessing the level of glucose with non-invasive sensors: optical, ultrasonic, electromagnetic, thermal, and others (15–17).

An additional filter with code from the Cooperative Patent Classification (A61B5/14532) and the keyword “non-invasive” was added to analyze the technological solutions associated with non-invasive sensors for glucose monitoring.

**Figure 8:**
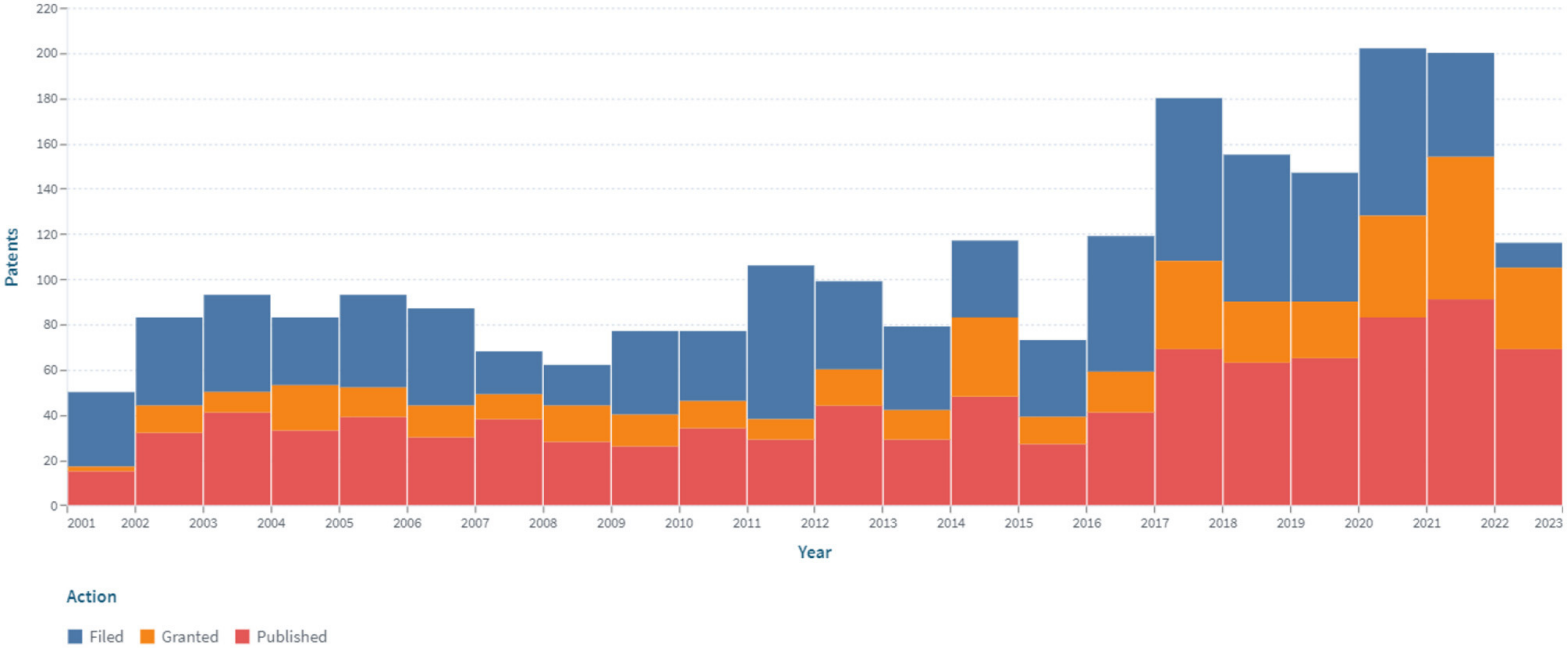
The dynamics of the patent landscape within the segment of non-invasive continuous glucose monitoring in the period 2000–2022.

The dynamics of the patent landscape in the segment of continuous non-invasive glucose monitoring are shown in Figure 8.

The dynamics of patent publications in the area of non-invasive continuous glucose monitoring have a positive trend. Nine hundred and ninety-five patents were issued, and 469 simple families are represented. In the area of non-invasive continuous glucose monitoring, the largest number of patents have been published in the United States (500; 50%), according to the PCT system (225; 23%), through the European Patent Office (91; 9%), and in China (69; 7%).

It should be noted that not all patented developments for non-invasive continuous glucose monitoring are available for purchase. GlucoWatch Automatic Glucose Biographer (Cygnus, Inc., USA), used reverse iontophoresis (patent WO9600110) and obtained FDA approval (75). However, the developers recalled the device from the market due to failures in measurement accuracy and reliability, as well as discomfort and skin irritations in users.

Near infrared spectroscopy is used in patented devices: the TensorTip Combo-Glucometer (CNOGA Medical), the Helo Extense (World Global Network), and the Wizmi (WEAR2B LTD).

The TensorTip Combo-Glucometer is disclosed in US Patent No. 10687739 (76). The device was developed by Cnoga Medical and approved by the CE (certified in Europe). The TensorTip Combo-Glucometer is pending FDA approval. The measurement method used in the TensorTip Combo-Glucometer is based on the method of photoplethysmography, which assesses changes in the state of the vessels inside the user's finger when illuminated with light of different wavelengths. The device is hybrid because it is calibrated to take into account the individual characteristics (skin color, skin thickness, etc.) of each person when measuring the blood sugar level by a traditionally invasive method using test strips. The TensorTip Combo-Glucometer requires constant recalibration.

There is evidence from clinical studies supporting the concept that the TensorTip Combo-Glucometer may be an effective additional alternative for glucose monitoring.

The TensorTip Combo-Glucometer was investigated in 76 patients with diabetes mellitus; 77 patients after heart surgery; 19 participants at home; and two post-marketing users (77). More than 98% of measurements in each study were reported to be in zones A (over 81%) and B (over 11%). The results demonstrate the compliance of the Combo glucometer with the reference methods.

In more recent investigations, the combined invasive and non-invasive TensorTip Combo-Glucometer (n = 100) results showed that 99% of glucose measurements were in zones A and B (91.1 and 7.8%, respectively), and MARD was 18.1% for non-invasive technology. The authors conclude that this technology allows for painless monitoring of glucose levels in people with diabetes (78).

Against the background of a standardized experiment with food in 36 patients, including healthy individuals as well as those with diabetes, the performance of the TensorTip Combo-Glucometer in comparison with the YSI Stat 2300 Plus at home was assessed. For the non-invasive TensorTip Combo-Glucometer instrument, 96.6 and 3.4% of results were detected in zones A and B. The authors conclude that the measurements were reliable with the Combo glucometer (79).

The results of the studies performed showed the effectiveness and accuracy of measurements using the TensorTip Combo Glucometer in patients with DM1 and DM2 and in healthy volunteers, and patients after heart surgery. The TensorTip Combo Glucometer is the “world's first” hybrid glucometer with both non-invasive and invasive components.

Near infrared spectroscopy is used in a medical sensor for non-invasive glucose monitoring in the Helo Extense device developed by World Global Network and disclosed in patent application WO 2022/076395 (80). The device has not been approved by the FDA yet and does not have the CE mark. The informativeness of HELO Extense device publications is limited. No clinical trial data were identified at this time.

The Wizmi device manufactured by WEAR2B LTD is disclosed in patents US 11129556, and EP 3975831 (81, 82). The device also uses near-infrared reflectance spectroscopy.

In a prospective, observational, controlled clinical trial in 32 healthy pregnant women, the safety and precision of non-invasive continuous glucose monitoring with the Wizmi sensor were compared with plasma glucose values (83). Of the 224 paired measures, 208 (93%) were in Zone A, and 16 (7%) were in Zone B, both of which were clinically suitable zones for the Clarke error grid analysis. The Wizmi device is safe to use, with overall good accuracy when compared to a gold standard reference of plasma glucose, according to the scientists' findings. Clinical trials of the Wizmi device are underway.

A medical sensor for continuous glucose monitoring developed by DiaMonTech and using laser detection is of interest. The device is under research and development. The DiaMonTech device was not cleared by the FDA. The device has received CE mark approval. The essence of the sensor is disclosed in patent EP 3623795 (84). The device operates in the infrared range and measures the glucose level using laser detection. The proposed system comprises a device to emit an exciting light beam and a probing light beam. The probing light beam is oriented so that it allows the analysis of many different materials with different optical densities. The mobile phone communication option allows you to check the information and take the necessary measures.

In studies using 100 volunteers, a non-invasive glucometer prototype that combines skin stimulation by a mid-infrared quantum cascade laser with photothermal sensing was examined. Results showed that non-invasive blood glucose analysis is practical and accurate enough to replace finger pricking and minimally invasive glucometers (85). Currently, the clinical trial “Feasibility study of blood glucose monitoring with the non-invasive medical device D-Base (DiaMonTech)” is active (86).

FiberSense is a continuous glucose monitoring device developed by EyeSense. WO 2022/199765 discloses a FiberSense device that measures fluorescence in a tissue surrounding the transport fluid by means of a fiber optic probe (87). A FiberSense CGM clinical trial is currently underway [clinical trial NCT05133973, “Feasibility Study of a Transdermal Continuous Glucose Monitoring (CGM) System in Diabetic Patients”] (88).

Interesting is the development of GlucoWise by MediWise, based on radio wave spectroscopy technology. In determining the glucose level, electromagnetic radiation with a frequency of 60 GHz is used, which is applied to the area of the skin between the thumb and index finger or to the earlobe. This technique is disclosed in patent US11298052 (89). GlucoWise is still in research and development.

The GlucoWise device was evaluated for glucose monitoring in a pilot study with healthy male volunteers (*n* = 10). For two volunteers, the results showed good agreement between invasive and non-invasive techniques. Data gathered from the other eight volunteers revealed abnormalities in the radio-frequency signal that could have been caused by stress and indicated hand motion and incremental holder sliding during the session (90).

Nemaura Medical introduces the SugarBEAT continuous glucose monitoring device to the market. Patent US 10092224 states that the device uses a reverse iontophoresis technique to extract glucose analytes from a patient's interstitial fluid (91). The patch system is in close contact with the patient's skin and contains electrochemical sensors. An electronic sensor collects readings and transmits them to a smartphone or SugarBEAT reader. Calibration requires a traditional finger-prick blood test.

Data from a clinical accuracy study of the SugarBEAT device in 75 patients with DM1 and DM2 are available. Over 12,000 pairs of data points, MARD was 7.96% for the 2-point calibration and 8.02% for the single finger prick calibration (92). Nemaura Medical, Inc. has received CE Mark approval for SugarBEAT.

OrSense Ltd. received CE approval for a continuous non-invasive glucose monitoring system by spectroscopy. This optical measurement technology is disclosed in EP 1292216 (93). This system was investigated in 23 patients with DM1 and DM2. A Clarke error grid analysis revealed that 95.5% of the data fell within the clinically acceptable A and B zones, and the mean relative absolute difference was 17.2%. The sensor was well-tolerated (94). However, the NBM-200G device has been discontinued by OrSense Ltd.

The non-invasive Symphony device is designed by Echo Therapeutics. Symphony includes a skin penetration tool, a transdermal sensor, a wireless transceiver, and data display technologies. Non-invasive glucose determination is performed using sonophoresis. The device is disclosed in numerous patents (including US 7963917 and US 8812071) (95, 96).

In critically ill cardiac surgery patients (*n* = 15), the Symphony continuous glucose monitor's accuracy has been investigated. An analysis of the glucose-error grid revealed that zones A and B were occupied by 99.6% of the measurements. MARD was at 12.3%. There were no known negative device impacts. The Symphony system, according to the involved scientists, can accurately measure glucose levels in the transdermal interstitial fluid of patients in cardiac surgery intensive care units (97).

Noteworthy is the development of GlucoTrack (GlucoTrack, Inc., formerly Integrity Applications). The device is disclosed in patent application WO 2022/020734 (98). The device combines three ways to measure blood glucose: ultrasonic, electromagnetic, and thermal. Integration of measurement data from three methods at the same time allows patients to get more accurate results. All these measurements are carried out using a miniature clip-on sensor, which is attached to the earlobe. GlucoTrack is a device that has received a CE Mark.

In recent years, data on the use of GlucoTrack has expanded significantly. Studies have been conducted on the accuracy of GlucoTrack in patients with DM1 and DM2, as well as on the influence of demographic factors.

For 2 days, GlucoTrack was compared to HemoCue in patients with DM1 and DM2 (*n* = 91). Clarke error grid analysis revealed that 96% of readings fall into acceptable zones A and B. The authors conclude that the high accuracy of GlucoTrack is achieved through the use of a combination of several technologies (99).

Of interest are also the GlucoTrack studies in patients (*n* = 172) with DM2 with various demographic characteristics related to age, sex, body weight, and the presence of a punctured earlobe. Studies conducted using the Clarke error grid analysis revealed that 97.6% of glucose readings were in zones A and B. The authors conclude that GlucoTrack measurements are independent of patient demographic profiles (100).

Lin et al. also investigated the accuracy of GlucoTrack according to the individual characteristics of patients (*n* = 114) with DM2 (101). The effect of such factors as duration of diabetes mellitus, HbA1c level, and smoking on the indicators of continuous glucose monitoring was assessed. 98.0% of glucose readings were found to be in zones A and B. Absolute relative difference values were independent of duration of diabetes, HbA1c, or smoking. The findings suggest that GlucoTrack's work is independent of diabetes duration, HbA1c levels, and smoking history.

The GlucoTrack medical sensor also exhibited stable performance in a trial of individuals (*n* = 27) with DM2, including those with prediabetes (102). It was found that 100% of the outcomes fell inside zones A and B (62.4 and 37.6%, respectively). MARD is 19.7% compared to YSI Stat2300plus and 17.5% compared to HemoCue.

Thus, the results of GlucoTrack studies showed its accuracy in both DM1 and DM2 diabetic individuals, independence from demographic factors (age, sex, body weight, and presence of a punctured earlobe), and individual characteristics of patients (duration of diabetes mellitus, HbA1c level, and smoking). GlucoTrack is also indicated for patients with prediabetes.

A fundamentally different approach for constant glucose monitoring is used by the developers of the Boydsense device. BoydSense Inc. is developing an exhalation analyzer using biomarkers to predict blood glucose levels. This technique is disclosed in EP 3830574 (103). Currently, clinical studies of the exhalation analyzer are under way. The clinical trial NCT05207020, “Development and Validation of the Blood Glucose Measurement Device by Air Analysis Expired (BOYDSENSE)” has a status of “Recruiting” (104).

Several studies have also investigated the potential of continuous monitoring of glucose in tear fluid. The inventors of the Akron Institute have developed diagnostic lenses for measuring glucose levels in tear fluid for patients with diabetes. If blood glucose rises, the lenses containing the boronic acid derivative change color. The invention is disclosed in patent CA 2774462, but the patent has now expired, and the development has not been widely used commercially (105). A glucose-sensitive contact lens was developed by Novartis and presented in the application WO 2003/075888 (106). It was also not commercialized.

Dongwoon Anatech has already received patent recognition for saliva glucose detection (D-SaLife) technology in South Korea, Japan, and China; an EP 3561507 patent application in Europe is pending (107). The sensor uses the glucose oxidase method.

The correlation and precision between the salivary glucose level measured with D-Salife and the capillary glucose level were examined in 114 people (108). Capillary glucose and D-Salife glucose had a direct association (*r* = 0.93), according to the regression analysis. According to error grid analysis, 33.3% of D-Salife were in zone B and 66.7% were in zone A. The authors came to the conclusion that the D-Salife performed well in accordance with ISO 15197. The company is planning further clinical trials of D-SaLife.

Other researchers have also proposed a system using salivary glucose screening. iQ Group Global has developed a medical sensor to detect salivary glucose. This organic thin film transistor is disclosed in patent application US 2020/0057020 A1 (109). The film contains glucose oxidase, which, upon contact with sugar in saliva, generates an electric current. It is measured using a special chip built on transistors. The mobile device receives a signal that is transferred electrically. The collected data is processed by the application and displayed on the screen. The sensor is disposable and made in the form of a strip. Data from clinical studies of this medical sensor were not revealed.

To control glucose, scientists also propose to analyze sweat. An electronic patch has been developed to monitor sweat glucose levels. The technology is disclosed in patent application KR20180002550 (110). The patch is equipped with sensors for humidity, glucose, pH balance, and temperature. When measuring the glucose level in sweat, the enzyme type sensor takes into account pH and temperature values to increase the accuracy of the results.

There are data from pilot studies of sensors for monitoring sweat glucose levels in patients (111). The sweat glucose levels detected by the sweat glucose sensors are consistently correlated with the blood glucose levels estimated by a commercial glucose meter, according to statistical analysis. Moreover, it should be noted that the researchers proposed integrating the sensor for monitoring sweat glucose with the transdermal drug delivery module.

## 4. Discussion

The studies conducted revealed a stable increase in the patenting of sensors for continuous glucose monitoring in the period 2000–2022, to the greatest extent since 2006. New devices can be expected to appear in medical practice in the coming years since patenting precedes the launch of products on the market. The greatest geographical distribution of development patenting was discovered in the United States and Europe, as was the protection of inventions under the Patent Cooperation Agreement. Leading development companies are Dexcom Inc., Abbott Diabetes Care Inc., Medtronic Minimed Inc., Roche Diabetes Care Inc., Roche Diagnostics Operations Inc., Roche Diabetes Care Gmbh, and Ascensia Diabetes Care Holdings Ag, among others.

It should be noted that continuous monitoring currently still complements, but does not fully replace, blood glucose control by other methods. The continuous glucose monitoring system gives a more comprehensive picture of the state of carbohydrate metabolism compared to the determination of glycated hemoglobin (HbA1c) and self-monitoring data using a glucometer, and enables the recording of glycemic fluctuations in detail during the day. In addition, in clinical practice, continuous glucose monitoring allows to assess the effect of nutrition, exercise, concomitant diseases, different doses of sugar-lowering or other drugs, and other factors on glycaemia and its variability. This allows initiating changes in patients' therapy and giving more targeted recommendations on diet compliance and exercise.

The role of continuous glucose monitoring in the estimation of asymptomatic and nocturnal hypoglycemia is also important, as it allows for the risk of their occurrence to be reduced, contributing to the improvement of the patient's glycemic profile.

### 4.1. Minimally invasive continuous glycemic monitoring

Positive dynamics of the patent landscape in the segment of invasive and minimally invasive continuous glucose monitoring have been established for the period 2000–2022.

The patented minimally invasive continuous glycemic monitoring devices (Guardian Real-Time System, Medtronic MiniMed, Dexcom G7, Dexcom Inc., FreeStyle Libre, and Abbott Diabetes Care) use an electrochemical mechanism for sensing glucose concentration. Different concentrations of glucose in interstitial fluid and blood can result in measurement errors by minimally invasive sensors. It should also be noted that the method of monitoring glucose in interstitial fluid in conditions of increased oxygenation is not applicable because of the possibility of erroneous indications of hypoglycemia associated with a decrease in blood flow in the area of sensor installation (compression, hypothermia). In addition, the use of a number of minimally invasive sensors is associated with the need to calibrate the device as well as regularly self-monitor glycemia with a glucometer in a number of cases. The above-described patented sensors for minimally invasive glucose monitoring are presented for purposes of illustration and not to underline limitations.

The effectiveness of minimally invasive monitoring using such tools as Dexcom G7 (Dexcom Inc., California, USA), Guardian Real-Time (Medtronic MiniMed, US), and FreeStyle Libre (Abbott Diabetes Care, USA) has been confirmed in a number of clinical studies in patients with DM1, including high-risk individuals, and DM2, with insulin injections and insulin pump therapy, and in children and older adults, and during pregnancy. Exercise and body mass index, glucose variability, should be taken into account when using sensors for continuous glucose monitoring. A decrease in HbA1c was detected upon using such monitoring.

Thus, studies have revealed a positive patent trend for sensors for minimally invasive glucose monitoring. This monitoring significantly supplements the data from invasive monitoring and, as a result, increases the informativeness of diagnostic studies due to numerous measurements taken during various patient conditions: before and after eating, during and after physical activity, against the background of glucose variability, in the dietary treatment of patients without diabetes, during fasting, and during pregnancy. The possibility of using minimally invasive glucose monitoring in different age groups should also be noted. So, according to the instructions, the FreeStyle Libre sensor is designed to measure the level of glucose in interstitial fluid in patients with DM aged 4 years. Medtronic also has a pediatric version of the Guardian REAL-Time System. The Dexcom G5 system is approved for adults and children 2 years of age and older.

At the same time, among the disadvantages of minimally invasive constant glucose monitoring are: patients' discomfort due to the introduction of subcutaneous sensors; the need for calibration using invasive devices in a number of cases; and the usability of sensors for a limited number of days due to biofouling.

It has to be mentioned that for more than 10 years, continuous minimally invasive glucose monitoring has been used in medical practice. These sensors are well-tolerated with the exception of possible individual side reactions, including discomfort, skin irritation, the development of erythema, bleeding (especially in patients with hemophilia), and allergic reactions.

According to the conducted studies, clinical use of devices has confirmed their efficacy, favorable safety profile, and ability to significantly improve patients' quality of life. Promising areas for improving sensors for minimally invasive continuous glucose monitoring are increasing the accuracy of results, optimizing calibration frequency, improving the convenience of wearing a glucose sensor on the patient's body, and optimizing the sensor's life.

### 4.2. Non-invasive continuous glycemic monitoring

Patenting non-invasive sensors for continuous glucose monitoring also displays a positive trend. However, in comparison to invasive and minimally invasive analysis, the numbers of the developed and implemented technologies in this area are lower.

Numerous techniques have been presented for non-invasive determination of glycaemia, including analysis of biological fluids from various parts of the body and organs (fluid of the anterior chamber of the eye, lacrimal fluid, saliva, sweat, interstitial fluid of the skin, etc.) and the composition of exhaled air. A number of devices have received CE mark approval (TensorTip Combo-Glucometer, Cnoga Medical Ltd.; SugarBEAT, Nemaura Medical; GlucoTrack, and GlucoTrack Inc.). However, these devices have not yet been approved by the FDA.

A clear advantage of non-invasive continuous glucose monitoring is the diversity of sensors using various glucose detection methods. However, it should be noted that some of the patented non-invasive sensors for continuous glucose monitoring are under investigation and are not yet on the market.

Continuous non-invasive monitoring has characteristics that make it highly desirable in the treatment of diabetes; this approach does not require implantation and does not cause adverse reactions of the organism to a foreign body. It should be noted that there is the possibility of error as a result of differences in skin pigmentation, hydration, intake of certain medications, and diverse parameters of the used body fluid. Moreover, a number of medical devices use non-specific methods to determine glucose, there is an effect of temperature on results, and there is no high correlation between blood glucose concentrations and glucose concentrations in the respective biological fluids. Nevertheless, continuous non-invasive glucose monitoring can also be used as an adjunctive method.

The analysis shows that non-invasive continuous glucose monitoring provides a large amount of glucose profile data and slows complications. It is necessary for patients and doctors, and it allows for timely glucose adjustment. The development of systems that account for the lag between the glucose concentration in the blood and interstitial fluid while requiring a minimum number of calibrations is promising.

### 4.3. Implementation of digital technologies and medical devices regulation (EU) 2017/745—MDR

According to the patent analysis conducted since 2006, a new trend in the advancement of continuous glucose monitoring has intensified, namely the implementation of digital technologies.

It is noted that early systems for continuous glucose monitoring included a sensor to estimate glucose concentrations, a transmitter that sent measurements to the receiver. Recent developments often use smartphones as receivers. This increases compliance with treatment because patients can quickly check glucose levels and their trends on their smartphones, which, as a result, optimizes the assessment of glycemic control (112).

Vettoretti et al. noted that potential users of low-cost CGM products include those taking part in weight reduction programs or athletes, for whom the CGM system may be useful for monitoring metabolism and making informed nutritional selections (113).

It should be noted that a number of non-invasive devices have received the CE mark, confirming that the products comply with European standards in the field of health, safety and environmental protection. It guarantees electrical safety and other issues mentioned in the regulations, but not health as such. In the European Union, there are certificates specifically for medical devices. Manufacturers could obtain certificates (MDD) in accordance with the Medical Devices Directive (Council Directive 93/42/EEC of June 14, 1993). However, in 2017, a European Union regulation on clinical trials and marketing of human medical devices [Regulation (EU) 2017/745] entered into force (https://health.ec.europa.eu/medical-devices-new-regulations/overview_en). It repeals Directive 93/42/EEC (MDD), which concerns medical devices, and Directive 90/385/EEC, which concerns active implantable medical devices, on May 26, 2021. The new Medical Devices Regulation (2017/745/EU) (MDR) harmonizes EU legislation according to technological advances, changes in medical sciences, and regulatory progress. In order to avoid destabilizing the market and to allow a smooth transition from the Directives to the Regulation, several transitional provisions have been implemented. Certain products certified under the Directives (with AIMDD or MDD certificates) may be placed on the market until May 26, 2024, and be available until May 26, 2025. During the transition period, manufacturers may obtain new MDR certifications under Regulation (EU) 2017/745. Compared to existing Directives, the MDR places greater emphasis on a life cycle approach that is clinically validated in terms of product safety. The MDR establishes stricter requirements for the designation of notified bodies and increases control and monitoring by national competent authorities and the Commission. MDR also enhances transparency by providing product information and publishing research results in the European Medical Devices Database (EUDAMED).

Examples of patented sensors for continuous glucose monitoring are presented in Table 1.

**Table 1:** Characteristics of patented sensors for continuous glucose monitoring.

**Device, company name, and country**	**Measurement technology and device location**	**Accuracy rate**	**Digital care for patients with diabetes**		**Status of the device**
			**Diabetes mobile health apps**	**Communication platforms (between patient and healthcare professionals)**	
**1**	**2**	**3**	**4**	**5**	**6**
Guardian Real-Time, Medtronic Minimed, US	Glucose oxidase-based electrochemical sensor; subcutaneous adipose tissue (the back of the upper arm, the upper buttocks).	Of the 3.941 paired measurements, 96% of the values fell within Zone A (61.7%) and Zone B (34.4%). Agreement between YSI and Guardian RT values tended to be closer when glucose values were in the mid-range, vs. high or low glucose levels (114).	A transmitter sends the measurements to a receiver. The Guardian Connect system is compatible with the mobile app. The Guardian Connect continuous glucose monitoring system allows patients to see glucose levels, trends, and alerts on their mobile device.	The CareLink system platform allows users to connect with care partners and healthcare professionals (HCPs) to enable care partner remote monitoring and HCP therapy optimization when connected to the internet via WI-FI or mobile data.	Approved FDA and commercialized.
Dexcom 7G, Dexcom Inc., US	Glucose oxidase-based electrochemical sensor; subcutaneous adipose tissue (the abdomen, the back of the upper arm, the upper buttocks).	With an 8.2% overall MARD for adults and an 8.1% MARD for children, 2 Dexcom G7 is the most accurate CGM system available (115).	Devices are compatible with the mobile app.	Dexcom Clarity allows patients to view trends, statistics, and day-by-day data and email them to healthcare professionals. Dexcom G6 and G7 let patients share their glucose levels with up to 10 people who use the separate Dexcom Follow app, giving patients an added layer of support.	FDA-approved, CE-marked, and commercialized. ^*^EU Certificate MDD for Dexcom ONE Continuous Glucose Monitoring System.
FreeStyle Libre Pro Flash Glucose Monitoring System, Abbott Diabetes Care, US	Amperometric electrochemical sensor; subcutaneous adipose tissue (the abdomen, the back of the upper arm).	Overall, the mean absolute relative difference was 12.3% for the comparison with the YSI reference. The median absolute relative difference shows that half of the time the system was within 10.1% of the YSI reference (116).	Mobile app. FreeStyle Libre Link allows patients to monitor glucose with their phones.	Mobile app. LibreLinkUp enables patients to share glucose data with friends and family and manage diabetes together. The LibreView platform enables patients to share their glucose reports with healthcare professionals between appointments in order to have more in-depth discussions about their diabetes management, as well as to provide clear, easy-to-understand reports and easy remote access.	FDA-approved, CE-marked, and commercialized
TensorTip Combo-Glucometer, Cnoga Medical Ltd., Israel	NIR photoplethysmography; finger	MARD of 14–17%; error grid analysis: 99% in zones A and B (91.1 and 7.8%, respectively) (78)	There is Singular mobile application for users' other devices of Cnoga Medical Ltd. (TensorTip VSM and TensorTip MTX).	There is Cnoga's doctor management platform for patients monitoring for users' other devices of Cnoga Medical Ltd.	CE-mark approved. Not cleared by FDA.
Helo Extense World Global Network, US	Near-Infrared Spectroscopy; finger.	No reliable data has been found.	The HELO Extense is compatible with the mobile app. There is a HeloAppStore, a dedicated site for apps developed by subject matter experts, exclusively for Helo device.	There is Lifelog, a partner platform powered by Helo data.	The FDA has not approved it. The CE-mark has not been received.
Wizmi, WEAR2B Ltd, Israel	Near-Infrared Spectroscopy; arm wrist.	Error grid analysis: 93% in zone A, 7% in zone B (83)	No data has been found.	No data has been found.	Clinical trials are underway.
D-Base, DiaMonTech, Germany	Laser detection; finger.	MARD: 11.3–12.1% Consensus error grid: 98.8% in zones A+B (85)	D-Base can be connected to a mobile phone.	No data has been found.	DiaMonTech has submitted a pre-submission application for D-Base to the FDA. CE-mark approved.
FiberSense, EyeSense, Switzerland	Fluorescence method; transdermal.	Clinical trials of the FiberSense device are underway.	FiberSense is compatible with the mobile app.	No data has been found.	The FDA has not approved it. The CE-mark has not been received.
GlucoWise MediWise, England	Radio wave spectroscopy technology; hand.	Clinical trials are underway.	A mobile application is being created.	Smart cloud technology is being created.	The FDA has not approved it. The CE-mark has not been received.
SugarBEAT, Nemaura Medical, US	Reverse iontophoresis technique; upper arm.	MARD was 7.96% for the 2-point calibration and 8.02% for the single finger prick calibration (92)	SugarBEAT is compatible with the mobile app.	There is a Beat Technology Platform. Transmitting data to healthcare professionals via a smartphone app, the technology will allow for medical conditions and chronic diseases to be monitored for better disease management or treatment.	CE-mark approved. FDA pending.
NBM-200G, OrSense Ltd., US	Occlusion spectroscopy, finger.	Error grid analysis: 95% in zones A and B (94)	No data has been found.	No data has been found.	CE-mark approved. Discontinued.
Symphony, Echo Therapeutics, US	Sonophoresis; transdermal.	Error grid analysis: 99.6% in zones A and B (97)	Symphony is compatible with the mobile app.	Echo glucose data will be displayed on the cloud service for ultimate user convenience and accessibility.	No reliable data has been found.
GlucoTrack, GlucoTrack Inc., Israel	Personal ear clip; ultrasonic, electromagnetic, and heat capacity techniques.	Error grid analysis: 96% in zones A and B (99)	The testing of the mobile app has been completed.	The testing of the cloud-based software has been completed.	CE-mark approved and commercialized.
Boydsense, BoydSense Inc., France	Identification, and quantification of volatile organic compound (VOC) patterns in human breath; exhalation analyzer.	Clinical trials are underway.	Public APIs for EMR, applications, and portal access are developing.	Public APIs for EMR, applications, and portal access are developing.	No reliable data has been found.
D-SaLife, Dongwoon Anatech, South Korea	Saliva glucose detection.	Clinical trials are underway.	D-SaLife is compatible with the mobile app.	No data has been found.	No reliable data has been found.
Saliva-based glucose test, iQ Group Global, Australia	Saliva glucose detection.	Data from clinical studies were not revealed.	Sensor is compatible with the mobile app.	No data has been found.	iQ Group files a pre-submission to the FDA for a saliva-based glucose test.
Biosensing device, Seoul National University, Institute for Basic Science, South Korea	Sweat-based glucose monitoring device.	Clinical trials are underway.	No data has been found.	No data has been found.	No reliable data has been found.

^*^EU Certificate MDD—certificate in conformity with European Directive 93/42/EEC on Medical Devices, submitted in the European Medical Database EUDAMED (accessed on July 18th, 2023).

^**^EU Certificates MDR—certificates in conformity with Regulation (EU) 2017/745, submitted in the European Medical Database EUDAMED have not been found (accessed on July 18th, 2023).

The progress in the field of sensors for continuous glucose monitoring offers a good opportunity for the further development of effective and high-quality care for diabetes patients. The validation data presented in the table for specific sensors give general overview on their important features, without an exhaustive list of performed studies. Overall, it is necessary to conduct additional studies of the accuracy of the increased number of various sensors in more patients, including different groups of risk and age, including reliable results for periods over 14 days. Straight-forward way to assurance of the unity and quality of results is to standardize the method validation procedure and the process of obtaining acceptance criteria for validation characteristics. The processes of obtaining criteria and conducting validation should constitute a mutually agreed-upon system based on a single scientific base.

Certification of systems for continuous glucose monitoring in accordance with Regulation (EU) 2017/745 is also required. The regulation contains a number of improvements: reinforcement of the criteria for designation and processes for oversight of notified bodies, improved transparency through a comprehensive EU database on medical devices, introduction of an “implant card” for patients, reinforcement of the rules on clinical evidence, reinforcement of the rules on clinical evidence, etc. (117).

Mobile phone users can access over 1,500 diabetes management apps, namely patient health tracking apps, applications that function as independent medical devices, and applications that use medical device data (118).

The conducted analysis confirms that the control of diabetes treatment is becoming increasingly digital through the use of both connected digital medical devices and telemedicine communication (119, 120). Digital healthcare is a relatively new direction, but it is actively developing. The high risk of complications in diabetic patients can be prevented using current methods of digital continuous glucose monitoring. Mobile applications and computer programs are important components of systems for continuous glucose monitoring.

To visually assess glucose changes, most digital technologies for continuous glucose monitoring utilize a graphical display of the daily glucose profile—studies with overlapping daily graphs. Digital technologies also provide the main glycemic control indicators. An individual glycemic profile of the patient is created based on the information uploaded to the cloud storage and allows a doctor or medical organization to systematize the data of all of their patients.

For example, using the cloud platform with the LibreView web interface, which allows doctors and patients to create and exchange reports, enables the medical institution to aggregate the glucose data of all patients associated with it, perform dynamic monitoring, and control. Di Molfetta et al. created algorithms for the digital diabetes platform LibreView for various groups of diabetics to ensure proper glucose monitoring data interpretation (121).

Sensors for continuous glucose monitoring help to improve diabetes management. Continuous glucose monitoring is increasingly using digital technologies. As a result, digital innovation is spreading and providing broad access to the latest information technology and resources.

## 5. Conclusions

Active patenting and implementation of minimally invasive and non-invasive sensors for continuous glucose monitoring have been identified. Continuous monitoring currently supplements, but does not replace, other methods of blood glucose control and allows for more efficient glycemic control assessment. The implementation of digital technologies is a promising direction in the development of continuous glucose monitoring for patients with diabetes mellitus.

Sensors for minimally invasive glucose monitoring are patented, registered, approved, and marketed more than non-invasive devices. The clinical use of minimally invasive sensors for continuous glucose monitoring confirmed their clinical effectiveness in both type 1 and type 2 diabetics, including young patients and pregnant women; on the background of a diet in patients without diabetes mellitus, during Ramadan; and their favorable safety profile and ability to significantly improve the quality of life of patients.

Since 2006, the implementation of digital sensors for continuous glucose monitoring, using smartphones as receivers and telemedicine communication, has intensified. It increases adherence to the treatment of diabetes and increases its effectiveness. The creation of systems, which include digital sensors for CGM, mobile applications, and web platforms for professional analysis of glycemic control and implementation of unified glycemic assessment principles in mobile healthcare, represent promising approaches for controlling glycaemia in diabetic patients.

The development and improvement of digital sensors is expected to effectively complement invasive monitoring data, contribute to achieving a high patients' quality of life, and reduce their mortality.

## Author contributions

OL, AA, and HW: conceptualization. OL and AA: methodology. OL: formal analysis and writing—original draft preparation. OL, ME, AB, AY, EP, AM, JH, AA, and HW: writing—review and editing. All authors have read and agreed to the published version of the manuscript.
